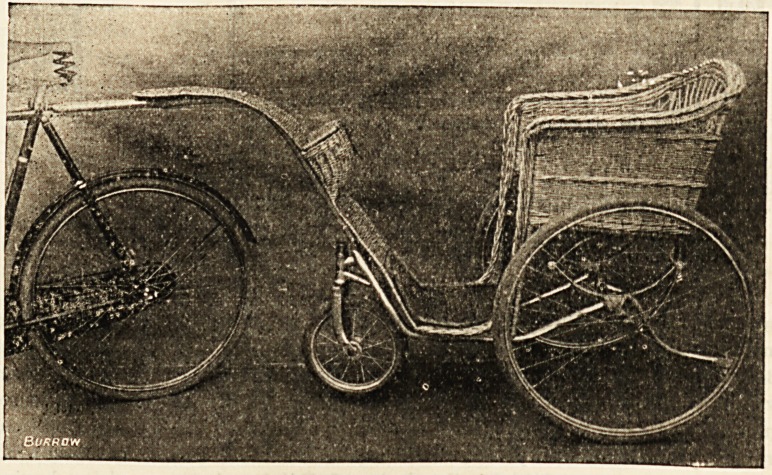# Practical Departments

**Published:** 1902-10-04

**Authors:** 


### PRACTICAL DEPARTMENTS.

### INVALID FURNITURE.

Probably it would be difficult to invent anything startlingly novel in the way of invalid furniture; carrying and wheel chairs, self-adjusting sofas, walking machines, bed-rests and tables, are to be had in such great variety at most of the shops where this kind of thing is sold. Providing there is money to pay for them, all the luxuries an invalid can possibly want are to be had in response to a message by telegram or through the telephone, and one cannot imagine anything more comfortable to aching bones or weary limbs than modern furniture for the invalid. One of the firms supplying such things is Messrs. Farmer and Marshall, of 12 Brompton Road, S.W. Their self-propelling chairs, one

Bed Rest.

Hospital "Merlin" Chair.

of which is a combined wheel and carrying chair, are light and strong; they have rubber-tyred wheels, and are moved along the floor by wheels worked by the patient's hands. A very nice arrangement is a chair that becomes a sofa when it is opened; it has soft cushions and arms, and goes on wheels. Light cane carrying chairs are very useful in a private house for moving the patient up and down stairs; one such is made to pack up into quite a small compass, and is very convenient for travelling.

Bed-rests and tables contribute largely to the comfort of the sick-room, and a rest that will take any angle is a necessity in cases where the patient quickly tires of one position. A very comfortable rest is one that is softly upholstered and has plenty of springs, and the arms can be folded back if they are in the way. Nearly as useful, if not quite, is a plain cane rest, also easily adjusted, on which any number of pillows can be placed; it can be had either with or without arms and side pieces to support the head of the invalid. Ten per cent. is allowed on purchases for which cash is paid, and furniture can be ordered by telephone.

### HOSPITAL FURNITURE.

Messrs. Maple are issuing a special catalogue of furniture suitable for hospitals and kindred institutions, and they invite medical men and others who are interested in the furnishing of such places to inspect their showrooms and see the various processes of manufacture. There is an almost endless selection of bedsteads in strong iron, with woven-wire spring mattresses or laths; the more elaborate ones, of course, have brass rails and knobs, while one kind is made with a bed-rest, also wire-woven, which moves up and down by a lever. All these are good and useful, and the prices seem moderate. In a spring mattress it is the middle which has to bear the greatest strain, and which consequently gives way, or "sags." To rectify this a special "Natsakka," or anti-sagging mattress, is now in use, in which the tension from every spring is concentrated on the middle; every spring thus bears an equal share of weight. Moreover, any part that has given way can easily be repaired. It has another advantage: everyone concerned with keeping beds and bedding in order knows how the ordinary wire-woven mattress collects and keeps the dust, and how difficult it is to get it out of the part that rolls over at each end. In the "Natsakka" there is nothing to roll over, and the difficulty of keeping it clean is therefore very much lessened. We notice a special bedstead for temporary use; the head and legs fold flat, and there is the same mattress as described above.

Space has frequently to be economised in hospital construction, and the "combination washstands and toilet-tables" made by this firm will be found especially useful in the fitting up of the staff bed-rooms. They have marble tops and tiled backs, and form also a chest of drawers. They do not take up much room, and are made in walnut, ash, or hazelwood. Bed-side lockers, bed-rests, and bed-tables, as well as sitting-room and board-room furniture, are made in many varieties, and the "enclosed washstands," with looking-glass in the lid, are most convenient in an office There are also "surgical lounges," carrying litters, merlin chairs, and many of the invalid appliances that are required in hospital or private cases. Catalogues can be had from Messrs. Maple, Tottenham Court Road, London.

### A TRAILING CAR FOR AN INVALID.

Those invalids who like to trust themselves to the care of a relative with a bicycle, rather than to the slow if steady

Bath-chair man, can have a "trailing car," and be drawn along at a rate which is certainly more exhilarating. A trailing car costs about as much as a good bicycle, and has all the fascination of tangent wheels, ball bearings, and pneumatic tyres, and its "parts" are stove-enamelled and nickel-plated. It has a little basket in front for carrying odds and ends, and altogether is very attractive to one who wants a bicycle ride without the fatigue of working the bicycle. The handle can be altered so as to turn the car into a common or garden Bath chair, but then the excitement ends, and you trail slowly up and down the sea front or the promenade like any other invalid. This car, as well as the more ordinary kinds of wheel chairs and other invalid furniture, may be had from Messrs. John Platter (Birmingham), whose London show rooms are at 34 Paternoster Row, E.C.

## Figures and Tables

**Figure f1:**
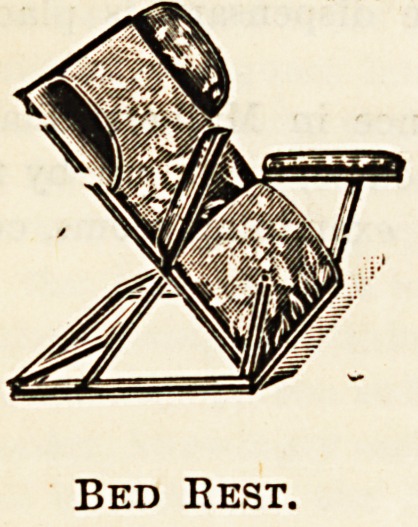


**Figure f2:**
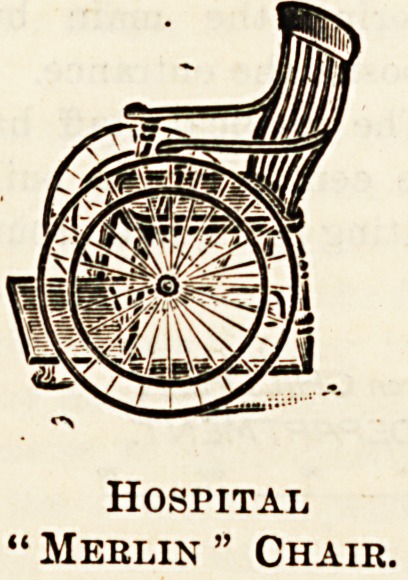


**Figure f3:**